# Retroviral superinfection resistance

**DOI:** 10.1186/1742-4690-2-52

**Published:** 2005-08-18

**Authors:** Micha Nethe, Ben Berkhout, Antoinette C van der Kuyl

**Affiliations:** 1 Dept. of Human Retrovirology, Academic Medical Centre, University of Amsterdam, Meibergdreef 15, 1105AZ Amsterdam, The Netherlands

## Abstract

The retroviral phenomenon of superinfection resistance (SIR) defines an interference mechanism that is established after primary infection, preventing the infected cell from being superinfected by a similar type of virus. This review describes our present understanding of the underlying mechanisms of SIR established by three characteristic retroviruses: Murine Leukaemia Virus (MuLV), Foamy Virus (FV), and Human Immunodeficiency Virus (HIV). In addition, SIR is discussed with respect to HIV superinfection of humans.

MuLV resistant mice exhibit two genetic resistance traits related to SIR. The cellular *Fv4 *gene expresses an Env related protein that establishes resistance against MuLV infection. Another mouse gene (*Fv1*) mediates MuLV resistance by expression of a sequence that is distantly related to Gag and that blocks the viral infection after the reverse transcription step. FVs induce two distinct mechanisms of superinfection resistance. First, expression of the Env protein results in SIR, probably by occupancy of the cellular receptors for FV entry. Second, an increase in the concentration of the viral Bet (Between-env-and-LTR-1-and-2) protein reduces proviral FV gene expression by inhibition of the transcriptional activator protein Tas (Transactivator of spumaviruses). In contrast to SIR in FV and MuLV infection, the underlying mechanism of SIR in HIV-infected cells is poorly understood. CD4 receptor down-modulation, a major characteristic of HIV-infected cells, has been proposed to be the main mechanism of SIR against HIV, but data have been contradictory. Several recent studies report the occurrence of HIV superinfection in humans; an event associated with the generation of recombinant HIV strains and possibly with increased disease progression. The role of SIR in protecting patients from HIV superinfection has not been studied so far.

The phenomenon of SIR may also be important in the protection of primates that are vaccinated with live attenuated simian immunodeficiency virus (SIV) against pathogenic SIV variants. As primate models of SIV infection closely resemble HIV infection, a better knowledge of SIR-induced mechanisms could contribute to the development of an HIV vaccine or other antiviral strategies.

## Introduction

Viral entry and replication is a complex process that involves multiple viral and host proteins. Many host gene products can interfere with virus infection at the cellular level (for a review, see: [1]). These proteins are encoded by variants of essential genes (that can not support viral infection), or represent true anti-viral factors (gene products whose main role it is to protect the cell from a productive virus infection). A special form of virus resistance is the capacity of cells to prevent a second infection by a virus that is closely related to the virus that has already established an infection. In most cases, virus-encoded proteins are responsible for this phenomenon, which is termed superinfection resistance (SIR) or viral interference. A simple form of SIR is receptor occupancy by viral Env proteins, preventing the binding of a second virus, but many additional mechanisms have been described. Although SIR is not restricted to retroviruses, it has been studied in depth for this class of viruses. This review deals with the molecular mechanisms of SIR at the cellular level in three retrovirus classes: simple retroviruses (here MuLV), spumaretroviruses (FV), and lentiviruses (HIV). The mechanisms and clinical consequences of HIV-1 superinfection in patients, which is defined as the reinfection of an individual with a second heterologous strain of HIV-1 [2], will also be discussed.

### Murine leukaemia virus

In the early 1950's, Gross identified a virus that could induce leukaemia in mice [3]. This discovery was quickly followed by the identification of additional leukaemia-inducing viruses, which led to the definition of the class of Murine Leukaemia Viruses (MuLVs). Although the list of MuLV related viruses is still expanding, most MuLVs can be divided into four classes: ecotropic, amphotropic, polytropic (sometimes called MCF viruses), and xenotropic. This classification is based on the type of host cell that is infected, based on the fact that the 4 classes use 3 different receptors. Ecotropic MuLVs can only infect murine cells, whereas polytropic MuLVs infect a broad host range of mammalian species including mice, albeit with variable efficiencies. Xenotropic MuLVs can infect many species, e.g. mink, rabbit, duck and human, but not cells of laboratory mice (reviewed in [4]). The polytropic and xenotropic viruses use the same receptor, Xpr1, also called Syg1. Polymorphisms in the Xpr1 protein determine the exact host range of the polytropic and xenotropic MuLVs. Ecotropic viruses use the amino acid transporter mCAT1 as their receptor, while the receptor for amphotropic MuLVs is the sodium-dependent phosphate transporter Pit2.

Cellular factors associated with MuLV restriction, have been studied extensively, whereby polymorphisms in the MuLV receptor genes were found to play a major role. Different cell lines were found to express functional variants of the ecotropic-, polytropic-, and xenotropic-MuLV receptors, which block infection by certain MuLV strains [5-7]. Proviral endogenous genes, like the mouse *Fv1 *and *Fv4 *gene products, can mediate restriction of MuLV replication by SIR associated mechanisms [8,9].

### *Fv1 *mediated resistance to MuLV infection

In 1967 the *Fv1 *gene was reported to be an important determinant of cell susceptibility towards MuLV infection [10]. Two common alleles for the *Fv1 *gene (*Fv1*^*b *^and *Fv1*^*n*^)present in prototypical mouse strains of BALB/c and NIH/Swiss, were found to interfere with certain classes of MuLVs (reviewed by [1,9]). Cells from NIH/Swiss mice, which carry the *Fv1*^*n *^allele were resistant to infection with the so-called B-tropic MuLVs. BALB/c mouse cells, which carry the *Fv1*^*b *^allele, were resistant to N-tropic MuLVs. In addition, a third class of MuLVs, the so-called NB tropic MuLVs, defined strains that can infect *Fv1*^*n *^as well as *Fv1*^*b *^expressing cells (all reviewed in [9]).

Substitution of defined regions within the N-tropic and B-tropic MuLV genomes by recombinant DNA cloning revealed that the *Gag *gene encoding the capsid protein CA determines the cell tropism. In particular, a single amino acid within the CA protein was identified to determine N or B tropism [11]. *Fv1 *mediated restriction occurs post-penetration and at or before integration of the proviral DNA genome [12], reviewed in [13].

**Figure 1:**
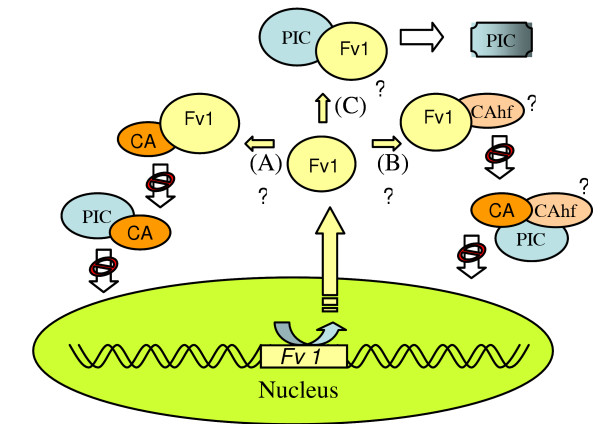
**Schematic of three possible interference mechanisms mediated by Fv1 expression**. Although the mechanism of Fv1 interference is still poorly understood and firm experimental evidence is lacking, several likely routes can be envisaged. Route A depicts the binding of Fv1 to CA, thereby restricting CA participation in the integration of the pre-integration complex (PIC) of MuLV DNA. In favour of this model, crystallographic studies recently suggested that a potential Fv1 binding domain exists in the MuLV CA [18]. Alternatively, if yet undetermined CA helper factors (CAhf) are needed during CA mediated integration of the PIC; binding of Fv1 to CAhf would prevent CAhf to assist CA during integration of the PIC (route B). A third possible route would involve direct binding of Fv1 to the PIC, thereby changing its conformation, and restricting it from further processing during CA-mediated integration (route C).

Cloning and sequencing of the *Fv1 *gene [8] showed that the *Fv1 *sequence is similar to the presumptive *Gag *gene of human endogenous retrovirus HERV-L (60% identity over a stretch of 1.3 kb). The *Fv1*^*n *^and *Fv1*^*b *^alleles differ by a few mutations, and in addition have a length difference of 19 amino acids at the C-terminal end. Gag proteins are known to interact tightly with each other, which is essential during virion assembly [1,14]. Possibly, interactions between the Fv1 Gag-like protein and viral Gag derived CA are involved in the Fv1 mechanism of resistance (for reviews, see [1,15,16]). CA has been suggested to act as a transport signal for the pre-integration complex (PIC) to facilitate import into the nucleus. The subcellular localization of the Fv1 product suggests it may affect virions on their way to the nucleus [17]. The most straightforward explanation of Fv1 mediated interference would be binding of Fv1 to CA in an allele specific way manner that alters CA binding to the PIC (fig. 1). The PIC could remain captured in the cytoplasmic compartment and thus not be able to migrate into the nucleus. However, a direct interaction between Fv1 and CA has never been demonstrated, although crystallographic studies recently suggested that a potential Fv1 binding domain exists in the MuLV CA [18]. Finally, a direct interaction of Fv1 with the PIC cannot be excluded, changing its conformation in such a way that it becomes non-functional (fig. 1). However, all mechanisms presented here to explain Fv1 restriction lack solid experimental evidence, and it should be noted that in the mouse genome there are hundreds of retroviral elements more closely related to MuLV than Fv1, and none of these restricts MuLV replication. A protein named TRIM5alpha has recently been characterized to restrict HIV-1 by an Fv1-like mechanism in primate cells. Restriction capabilities of TRIM5alpha vary amongst primates, so that rhesus monkey TRIM5alpha restricts N-tropic MuLV and HIV-1, but not B-tropic MuLV, while human TRIM5alpha restricts N-tropic MuLV, but not B-tropic MuLV or HIV-1 (reviewed in [19]). The ability to restrict HIV-1 is determined by a single amino acid in the C-terminal SPRY domain of TRIM5alpha [19]. As for Fv1 restriction of MuLV, TRIM5alpha targets the HIV-1 CA protein. Several mechanisms have been proposed for TRIM5alpha restriction, including binding and trapping of incoming virus, interference with uncoating, inhibition of SUMOylation (and thereby interfere with intracellular trafficking of the PIC), and targeting the incoming particle for proteasomal degradation whereby TRIM5alpha transfers the ubiquitin molecules to CA (reviewed in [1,9]). Elucidating the way by which TRIM5alpha restricts retroviruses might also shed light upon the mechanism of Fv1 restriction.

### *Fv4 *mediated resistance to MuLV infection

In 1975, Suzuki described the discovery of a new resistance gene, *Fv4*, in the G strain of laboratory mice [20,21]. The *Fv4 *gene was also identified in Asian wild mouse species [22]. Genetic mapping studies located the *Fv4 *gene on chromosome 12 [23]. There are two alleles at the *Fv4 *locus: the *Fv4*^*r *^resistance allele is dominant [20,21]. A first clue about the nature of the *Fv4 *gene came with the identification of MuLV Env related proteins in Fv4 resistant cell lines [24], which suggested an *Env*-like sequence for the *Fv4 *gene. Using an *Env*-specific probe, a 5.2 kb fragment of the *Fv4*^*r *^was cloned that contained part of the *Pol *gene, the entire MuLV *Env *region and the 3' long-terminal repeat (LTR) of an ecotropic MuLV [25,26]. Sequence analysis revealed that *Fv4 Env *encodes a surface (SU) and transmembrane (TM) Env domain that closely resembled (>90%) the homologous *Env *sequences in the unusual ecotropic MuLVs found in Asian wild mice [22]. Transgenic mice carrying the *Fv4 *gene showed complete resistance to ecotropic MuLV infection [27]. Moreover, transplantation of a certain percentage of Fv4 resistant bone marrow cells into the bone marrow of Fv4 susceptible mice strains induced full resistance against MuLV infection [28]. Although Fv4 mediated resistance has been demonstrated in different experimental systems, the underlying molecular mechanism remains unclear. As described earlier, Env-receptor interactions mediate retroviral entry into the target cell. Therefore, *Fv4*^*r *^mediated resistance has been suggested to rely on Fv4 Env binding to the MuLV receptor, which prevents exogenous MuLV infection. Substitution of the complete *Fv4*^*r *^*Env *gene in MuLV clones abrogated viral entry, indicating that the protein is defective [29]. The defect was attributed to a single amino acid substitution in the fusion peptide of the *Fv4*^*r *^Env protein, which when artificially introduced into an MuLV clone led to an Env protein that was able to bind to the cellular receptor, and was incorporated into virus particles at normal levels, but was incapable of promoting fusion and viral entry [30].

### Fv4 and mCAT1 interactions

**Figure 2:**
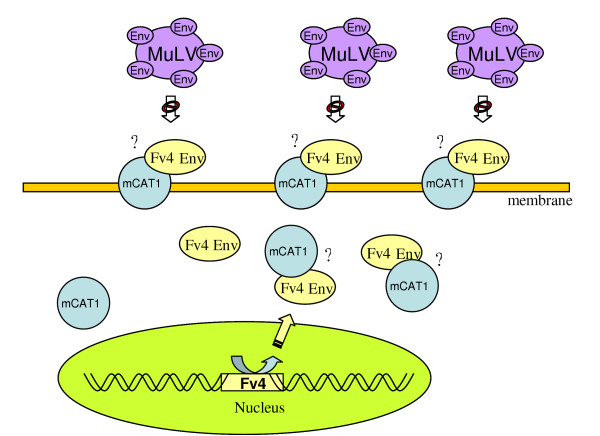
**Schematic of Fv4 mediated interference of MuLV infection**. Fv4 expression results in sustained levels of Fv4 Env proteins in the cytoplasm. Binding of the mCAT1 receptor by Fv4 Env proteins, either in the cytoplasm or at the cell surface (the exact location of interaction is unresolved, which is represented by question marks), prevents MuLV Env to interact with mCAT1, as either the receptor is already occupied by Fv4, or it cannot reach the cell surface when bound to Fv4 in the cytoplasm.

Sequence analysis of the ecotropic MuLV receptor showed it to be a cationic type 1 amino acid transporter (mCAT1) [31,32]. Comparable expression patterns of *mCAT1 *mRNA have been described for different tissues of *Fv4*^*r *^congenic MuLV-resistant (C4W = BALB/c-Fv-4W^r^) and -susceptible (C3H/HeMsNrs and C56BL/6) mice strains. However, recombinant F-SU/GFP, consisting of the SU domain of Friend MuLV and the GFP protein, was unable to stain most *mCAT1 *mRNA expressing tissues of the C4W (*Fv4*^*r*^) mice strain [33], suggesting that either an intracellular downregulation of the receptor has occurred, or that the receptor is blocked at the cell surface by the Fv4 gene product (fig. 2).

Altogether, these data strongly suggest that *Fv4*^*r *^interferes with MuLV infection by masking of the MuLV receptor through binding of Fv4 Env. Two other mouse interference genes, named *Rmcf1 *and *Rmcf2*, also cause MuLV resistance by Fv4-related interference mechanisms [34,35]. Crosses between an *Rmcf1 *resistant mouse strain and an *Rmcf1 *lacking mouse strain revealed that inheritance of *Rmcf1 *resistance correlated with the inheritance of an endogenous MCF virus *Env *gene. The *Rmcf2 *gene also encodes an Env glycoprotein, and its expression blocks infection by polytropic MuLVs [35].

### Foamy viruses

In 1950 a new type of retrovirus was isolated from cell cultures derived from monkey kidneys. Foamy viruses (FV) were named after the characteristic foam-like effect they induce in cell culture. FVs are considered to be harmless in experimentally infected animals. The various unique features of FVs concerning their replication led in 2002 to the establishment of a new, distinct retroviral subfamily: the *spumaretrovirinae *(reviewed in [36]).

The genomic structure of FVs indicates that these viruses belong to the more complex retroviruses. The FV genome transcribes, besides *Gag*, *Pol *and *Env*, two major mRNA's from an internal promoter near the 3'end of the genome (reviewed in [36]): a DNA binding protein called Transactivator of spumaviruses (Tas), and the 60 kDA Bet protein. Tas is involved in the switch from latent to lytic virus replication, while Bet has a negative regulatory effect upon the internal promoter [37,38]. Furthermore, Bet can inhibit the APOBEC3 family of antiretroviral proteins [39], and mediates SIR [40].

### FV Env mediated SIR

As retrovirus entry depends on the interaction of the SU domain of Env with the target receptor, down-regulation of such a receptor would be a plausible mechanism for SIR. To date no receptor has been identified for FV. It has been proposed that a pH-dependent fusion process mediates foamy virus entry [41]. To investigate FV superinfection, Moebes and colleagues [42] tested whether overexpression of the FV Env protein induced SIR by downregulation of the putative receptor. Indeed, BHK-21 cell lines containing a stably transfected *Env *gene were completely resistant to infection with FV vectors that use FV Env for entry.

Deletion analysis of the FV Env protein showed that several properties of Env are needed to induce SIR: membrane anchorage of Env extracellular domains, efficient cell surface transport of the Env protein, and correct processing of the Env subunits [43]. So, in contrast to MuLV Env, secretion of FV Env is not sufficient to induce SIR.

A recombinant FV SU-Ig protein and FV Env expressing cell lines were constructed to study FV Env binding to the surface of target cells [44]. The receptor for FV is still undetermined, and it is possible that general features on the membrane surface, like for example glycolipids, mediate FV entry. This would explain the broad infection range of FV on mammalian and non-mammalian cells [45]. However, the binding experiments suggested that SIR by FV Env is similar to SIR by other retroviruses, whereby high expression of FV Env in stably transfected cell lines led to a complete resistance to FV SU-Ig binding and FV permissiveness, and low expression of FV Env led to a decreased susceptibility to infection and a lowered FV SU-Ig binding [44].

Concluding, the expression of FV Env proteins establishes resistance against FV superinfection. Moreover, FV Env proteins induce SIR at the cell surface, which suggests down-regulation of cell surface FV entry mediators. However, the exact underlying mechanism of SIR remains unclear.

### Bet mediated resistance to FV superinfection

Chronically infected FV cells, which are characterized by reduced production of Tas, are found to express predominately ΔHFV, a distinct proviral form of FV [46,47]. A persistent but latent infection is common in FV infected animals (reviewed in [48]). ΔHFV contains a 301-bp deletion in the Tas gene, which is spliced out from the pregenomic RNA [46]. Interestingly, ΔHFV seemed to interfere with FV infection [38]. This interference strongly correlated with the number of integrated ΔHFV copies [38]. ΔHFV constructs with a defective Bet gene were unable to interfere with FV infection [38], suggesting that Bet is involved in SIR. Normally, ΔHFV transfected cells contain stable levels of Bet mRNA and protein, and Bet is the major viral protein expressed in chronically infected cells [38].

The establishment of a Bet-expressing cell line confirmed a Bet-mediated induction of SIR [40]. Interestingly, Bet-induced SIR is unlikely to be mediated by Env-directed down-regulation of the FV receptor, as no Env mRNA or proteins were detected during the early phase of ΔHFV interference with FV infection [38]. In addition, Bet^+ ^cells did not prevent infection by a GFP-MuLV vector containing a ΔHFV envelope construct whereby the cytoplasmic tail of the transmembrane part is derived from MuLV [40]. As this vector contains the HFV envelope surface and TM domains, it must use the FV receptor to gain access to the Bet^+ ^cells.

**Figure 3:**
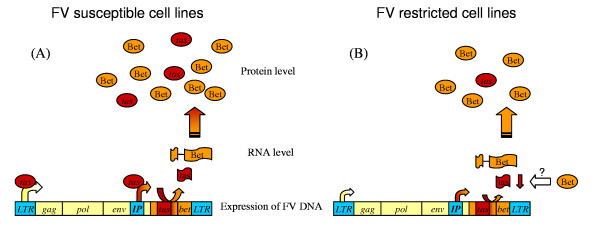
**Expression of Bet and Tas in FV susceptible and restricted cell lines**. FV susceptible cell lines containing abundant concentrations of Bet and low concentrations of Tas are still able to enhance the LTR and internal promoter (IP) (panel A). Restricted FV cell lines are associated with reduced LTR and IP activity (panel B). Bet-mediated inhibition of IP activation results in reduced concentrations of Tas and consequently further inhibition of IP activity. The underlying mechanism of Bet-mediated inhibition of IP could be a negative control on transcription or translation of the *Tas *gene as indicated by a question mark.

Infection of Bet^+ ^and Bet^- ^cells by FV resulted in 3–4 fold lower titres in the Bet^+ ^cells [40]. As proviral DNA was able to integrate into the host genome, Bet possibly interferes with FV replication during transcription of the provirus, although the lower levels of FV in Bet^+ ^cells could suggest an additional effect upon viral entry. Foamy viruses contain an internal promoter that drives transcription of *Bet *and *Tas *mRNA (reviewed in [36]). The transactivator Tas activates both the LTR and internal promoters by direct binding [37]. Bet and Tas are produced from overlapping reading frames and mediate opposite effects on FV replication (fig. 3). Cell lines chronically infected with FV contain abundant levels of the negative regulator Bet, low levels of structural proteins and of the transactivator Tas, and a high ΔFV load [37,38]. Increasing the level of Tas by transfecting latently infected cells with a Tas expression vector triggered FV replication and cell lysis [37]. Thus, Bet reduces FV replication by inhibition of Tas expression, which in turn reduces internal promoter activity. The exact mechanism by which Bet inhibits Tas expression is not clear. Bet protein could stimulate splicing of its own mRNA, which consequently would alter Tas RNA levels. Other possibilities are Bet-mediated inhibition of Tas RNA transport or decreased stability of Tas RNA. It seems unlikely that Bet prevents Tas expression by stimulation of promyelocytic leukaemia protein (PML), the only known inhibitor of Tas [49], as significant amounts of PML were unable to prevent FV replication [50].

### HIV superinfection resistance

To date an estimated 40 million people worldwide are infected with the Human Immunodeficiency Virus (HIV), classified as a lentivirus within the class of retroviruses. HIV is associated with the development of Acquired Immune Deficiency Syndrome (AIDS). Two main virus types exist, HIV-1 and HIV-2, of which HIV-1 infection is the most important cause of AIDS.

Like all other retroviruses, the HIV virion contains two copies of an RNA genome that is encapsulated by CA and Env proteins. The Env glycoproteins gp120 and gp41 mediate viral entry by interacting with CD4 molecules on susceptible cells. The CD4 receptor is a type 1 transmembrane glycoprotein and is mainly found on primary T lymphocytes, dendritic cells and macrophages. Interaction of gp120 with CD4 induces conformational changes in the Env protein structure, which enables Env to interact with a coreceptor, such as the CCR5 or CXCR4 chemokine receptor, which leads to HIV entry into the target cell (reviewed in [51]). Several host factors have been identified that interfere with early steps during entry or replication of HIV-1, e.g. APOBEC3G/CEM15, Lv1, Lv2, and TRIM5alpha (for a review, see: [16]). Additional mechanisms by which an initial virus can inhibit entry or replication of a second virus will be discussed below.

Since the identification of the AIDS virus, various strategies have been proposed to prevent the spread of HIV infection. The underlying mechanisms of SIR in HIV-infected cells are of particular interest for the development of novel antiviral approaches related to SIR. However, as a caveat, we note that several studies describe the occurrence of HIV superinfection in patients. The next sections will describe the current understanding of the underlying mechanisms of SIR by HIV-1.

### CD4-mediated resistance to HIV superinfection

One of the major characteristics of HIV-infected cells is down-modulation of the CD4 receptor [52-54]. To date three viral HIV proteins; Vpu, Env, and Nef have been identified that mediate CD4 down-regulation by distinct mechanisms (reviewed in [55,56]), indicating the importance of CD4 down-regulation for HIV infection. As receptor down-modulation is a simple way of preventing a second viral infection, and a method that is successfully used by other retroviruses, CD4 down-modulation was initially assumed to be the main SIR mechanism in HIV infection.

All primate lentiviruses, HIV-1, HIV-2 and Simian Immunodeficiency Virus (SIV), encode the Nef protein (reviewed in [55]). Nef binds directly to a di-leucine-like motif in the cytoplasmic domain of CD4. Nef is able to bind different members of the adaptor proteins (AP-1, AP-2, AP-3 and AP-4), which contain distinct transport signals. Simultaneous binding of Nef to CD4 and AP-2 at the cell surface induces endocytosis of CD4. In addition, Nef binding of AP-1 and AP-3 in the trans-Golgi network may mediate trafficking of newly synthesized CD4 directly to lysosomes. Stable transfection of the SIV *Nef *gene in a CD4^+ ^T cell line reduced cell surface-expression of CD4, and rendered the cells resistant to subsequent HIV-1 infection [57]. As HIV-1 transcription was not inhibited in these cells, the authors speculate that the inhibition of superinfection in this model system is due to Nef-induced CD4 down-modulation. Besides, a clonal HIV-1 containing T cell line with down- regulated CD4 expression is also resistant to HIV-2 superinfection [54]. HIV-2 infected cells do not seem to resist subsequent HIV-1 infection, which may be explained by the inability of HIV-2 to induce CD4 down-modulation.

In contrast to Nef, Env and Vpu mediate CD4 down-modulation by preventing the intracellular transport of newly synthesised CD4 molecules (reviewed in [56]). Binding of CD4 by the Env precursor protein gp160 in the endoplasmatic reticulum (ER) triggers the formation of aggregates, which block further CD4 transport to the cell surface. In addition, Vpu mediates CD4 down-modulation by directing newly synthesised CD4 to proteosomes for degradation. Among the immunodeficiency viruses, Vpu is encoded nearly exclusively by HIV-1. Vpu has been suggested to redirect CD4 trafficking by acting as an adaptor between CD4 and the h-βTrCP protein that is a key connector in the ubiquitin-mediated proteolysis machinery. Restriction of Vpu mediated CD4 down-modulation either by inhibition of the proteosome activity or mutation of putative ubiquitination sites in the CD4 cytoplasmic domain supports this hypothesis.

The most important physiological purpose of CD4 down-modulation is likely not to resist superinfection, but rather to increase viral replication and to promote the release of progeny virions [57,58]. Reduction of CD4 cell surface-expression results in particles with less CD4 and more Env molecules, which probably eases their release from the cell. When using HIV-1 variants with different coreceptor usage obtained from patients, it was found that down-modulation of CD4 was not associated with CCR5-using viruses that are present early in infection, but were characteristic of CXCR4- or CXCR4/CCR5-using viruses that are mostly seen later in infection during the onset of AIDS [59]. In line with this, Lusso *et al. *[60] found that a macrophage-tropic, non-cytopathic strain of HIV-1 that did not down-regulate CD4, did also not resist subsequent superinfection with a cytopathic HIV-1 strain in a CD4+ T-cell clone (PM1) susceptible to a wide variety of HIV isolates. Furthermore, *Nef*-genes from AIDS patients were far more efficient in down-regulating CD4 than *Nef*-alleles from asymptomatic patients [58]. Together, these results raised a question whether CD4 down-modulation *in vivo *is a significant cause of SIR in HIV-1 infection.

Additional questions about the relevance of CD4 down-regulation come from analysis of the kinetics of CD4 down-modulation in HIV-infected T cells. CD4 down-regulation starts two days after infection and just a few hours before the cells are committed to die (reviewed in [56]). This leaves only a small time span in which CD4 down-modulation of infected transformed T cell lines may interfere with HIV superinfection. Moreover, down-modulation of CD4 in primary T-lymphocytes occurs even later. The half-life of HIV-infected cells in patients has been estimated at 1 to 2 days. Volsky and colleagues [61] demonstrated SIR to be established relatively early between 4 and 24 hrs after primary HIV-infection. Thus, the kinetics of CD4 down-modulation would imply that the established resistance to HIV-1 superinfection is not mediated by CD4 down-modulation. Indeed, HIV-1 SIR has been demonstrated to occur independently of CD4 down-modulation as will be discussed hereafter.

Co-receptor down-regulation could be an alternative SIR mechanism. However, down-regulation of CXCR4 was not observed in culture, and although chronic infection with CCR5-using viruses abrogated CCR5 expression, the effect on superinfection was not tested [59]. A single study suggested that CCR5 down-modulation in an HIV-2 infected cohort of Senegalese women protected them from HIV-1 superinfection [62].

### CD4-independent mechanisms contributing to HIV SIR

A few studies have shown cellular resistance to HIV superinfection by mechanisms unrelated to CD4 (reviewed in [63,64]). Volsky and colleagues [61] demonstrated SIR in HIV-1-infected T cells that still expressed substantial levels of CD4. Moreover, non-functional HIV-1 mutants and HIV-1 mutants that could only bind CD4, but not enter the T-cells, did not restrict superinfection of HIV-1 in these cells. The mechanism was HIV-1 specific, as the cells could be infected by other (retro)viruses, indicating that the results could not be explained by a general block of virus replication. HIV-1 mutants that encode inactive *Vpu*, *Vpr *and *Nef *genes were fully active in SIR, ruling out these genes as contributing to HIV SIR.

Another study demonstrated CD4 independent SIR mechanisms in cells infected with a non-producer HIV mutant [65]. CD4 down-modulation in these F12-HIV-infected cells did not change their susceptibility to a challenge HIV strain. However, SIR was established by inhibiting the replication of the superinfecting HIV strain. An additional study evaluated SIR in cells transfected with distinct vectors containing a particular HIV protein [66]. The F12-HIV genes *Gag*, *Vif *and *Nef *were all found to alter replication of the superinfecting HIV-1 strain. Moreover, expression of Nef established complete resistance against the challenge by inhibiting HIV-1 replication at a late stage. Nef mediated inhibition of viral replication has been associated with interference of Gag processing by preventing the cleavage of the p41 Gag precursor protein into p17 (MA) and p24 (CA) [67]. Moreover, the disturbed processing of Gag has been correlated with an altered sub-cellular distribution of F12-Nef compared to the wild-type Nef protein.

### CD8 T-cells and HIV superinfection resistance

In animals, antiviral effects, either to the initial or to a second viral infection, are in large mediated by the immune system, making superinfections of animals greatly different from SIR in cells. A 100% effective SIR mechanism could prevent superinfection of a given cell in an animal, but a second virus could infect another, non-infected cell, leading to superinfection of the animal, but not to superinfection of the already infected cell. Neutralizing antibodies restrict re-infection of cells from seropositive donors in culture [68], and cytokines induced by the first viral infection can have a negative effect on subsequent infections [69]. An important immune-mediated inhibition of viral replication is exerted by non-cytotoxic CD8+ T-cells. These cells belong to the innate immune system and were found to suppress HIV-1 replication in CD4+ T-cells by a non-cytotoxic mechanism mediated by a soluble antiviral factor, provisionally named CAF [70] (for reviews see: [71-73]). Until now, the identity of CAF, short for CD8+-cell antiviral factor, has not been resolved, but it suppresses transcription of viral RNA [74,75], is found in both healthy persons and in asymptomatic HIV-1 infected patients [76], can be inhibited by protease inhibitors [77], and strongly suppresses HIV-1/HIV-2 superinfection in culture [78], and as such is included in this review. The mechanism is not virus or species specific, and is also operational *in vivo*. It has been found in HIV-2 infected baboons [79], and in FIV (feline immunodeficiency virus)-infected cats [80]. HIV-2 infected PBMC from pig-tailed macaques, however, can be superinfected with another strain of HIV-2 *in vitro *in the presence of CD8+ T-cells [81]. Furthermore, 80–100% of chimpanzees experimentally infected with HIV-1 could be superinfected after 8 to 64 months with a same or different viral subtype despite a fully functional immune system (reviewed in [82]).

Besides CAF as soluble factor, the studies by Locher *et al. *[79] and Chun *et al. *[83] suggest that contact between CD4+ and CD8+ cells is important for inhibiting viral replication, including HIV-1 superinfection. During disease progression, the anti-HIV effect of the CD8 T-cells is gradually lost [76,84], as is their ability to suppress superinfection [78], which is probably due to a functional impairment of the (HIV-specific) CD8+ cells in the AIDS phase [85].

### HIV superinfection in vivo

HIV-1 can be classified into three distinct groups based on genome sequences; M (major group), O (outlier group) and N (non-M/non-O group), which can be further subdivided into different subtypes (reviewed in [86]). The M group represent the major HIV-1 strains responsible for the worldwide spread of AIDS, and encompasses at least 10 distinct subtypes. The O group represents a minority of the HIV-1 strains and is found in approximately 2–5% of HIV-1-infected individuals in West and Central Africa.

In the late 80's and early 90's of the last century, a number of primate models demonstrated the possibility of HIV-1 superinfection *in vivo*, a phenomenon that was later also described in humans (reviewed in [2]). Several papers report HIV-1 dual infections as co-infections and not superinfections, as successive infection with two different viruses is often difficult to prove due to limited sampling. It is likely that in a patient, a second virus infects cells that are not infected by the resident virus. Superinfection of HIV-1 in humans can be classified as intra-subtype-, inter-subtype- or inter-strain (M/O)- superinfection. Three studies reported HIV-1 group B-infected individuals to be infected by a distinct subtype B virus [87-89]. HIV-1 subtype B superinfection occurred in two cases in the absence of any antiviral drugs, and in one case during treatment interruptions. A multiple drug-resistant virus was the initial infecting clade B virus in two patients. In all cases, the appearance of the second virus resulted in a decline in CD4+ T-cell counts and an increase in HIV-1 plasma levels. Three cases of HIV-1 superinfection with different subtypes of HIV-1 group M, all with subtype B and CRF01_AE, have been reported so far (reviewed in [2]). A HIV-1 triple infection was recently reported in a Dutch patient practising unsafe sex [90]. One year after the original infection with a subtype B strain, this patient was superinfected with a second subtype B strain, and again a year later another superinfection occurred, this time with subtype CRF01_AE. Only the second superinfection resulted in an increase in viral plasma load and a decrease in CD4+- cell counts and was accompanied by flu-like symptoms. Another triply infected individual, this time from Tanzania, was infected with a subtype C strain and two divergent subtype A strains [91]. However, in this patient it was not clear whether the triple infection was the result of superinfection or of simultaneous infection.

Thus, different HIV-1 group M subtypes are able to establish superinfection resulting in all cases in increased disease progression. Several studies have identified individuals who are dually infected with two distinct HIV-1 strains. In 1999, a dual M/O infected Cameroonian patient was identified [92], followed by five additional M/O dually infected individuals [93,94], and one O/M superinfected patient [95]. Apart from these anecdotal reports, several studies have attempted to study superinfection rates in cohorts of highly exposed individuals. In two cohorts, and in one study involving 14 HIV-seroconcordant couples, no evidence for superinfection was found [96-98], but several other studies report significant rates of superinfection in recently infected individuals. Three cohorts of intravenous drug users showed a 2.5–5% incidence of HIV-1 superinfection [99-101]. A 19% incidence was scored in a cohort of female sex workers within three months of primary infection [102]. However, the latter superinfections were transient, and no evidence of dual infection was seen after 24 months of follow-up [102]. A transient subtype B superinfection was also apparent in one of the intravenous drug users [99]. The incidence of HIV-1 superinfection is probably increasing as more people become infected, as this enhances the chance of meeting an already infected partner.

Viral recombinants, which are an indicator of superinfection on a cellular level, have been reported from the beginning of the epidemic. It has been suggested that recombination is an important viral evolutionary strategy for HIV, and may be considered a key aspect of viral reproduction, so-called "viral sex" [103]. Recombinants provide strong evidence that cellular SIR is not absolute, i.e. in patients some cells are superinfected at some frequency.

The occurrence of HIV-1 superinfection in humans raises questions about the possibility of developing an effective HIV-1 vaccine, both because of the obvious lack of protection of an already infected individual to a second infection (reviewed in: [82,104]). Nowadays, at least 15 circulating recombinant forms have been recognized within HIV-1 group M [2], and many more exist in individual patients. Fang and colleagues [105] described an A/C recombinant HIV-1 virus that was formed in a female sex worker, who was superinfected with HIV-1 subtype C after primary infection with HIV-1 subtype A. The development of new HIV-1 recombinants could also quickly alter various properties of HIV-1, such as cell tropism, viral pathogenicity, antiretroviral drug susceptibility and disease progression.

The studies described here clearly demonstrated HIV-1 superinfection in humans both with different HIV-1 strains and with closely related HIV-1 subtypes. In an asymptomatic patient, a large reservoir of uninfected cells is available for infection by a second virus, as only 1:2,500 to 1:100,000 CD4 cells are estimated to be infected by HIV [106,107]. During disease progression, substantially more virus is produced, and more CD4+ cells become infected [107]. Thus, in the later stages of HIV infection, when both viruses produce a significant amount of progeny, uninfected cells can become dually infected with both virus strains, enabling the formation of recombinant forms. In other cells of the same organism, SIR might be operational and prevent a second infection. Indeed, in splenocytes of two HIV patients, an average of three to four HIV-1 proviruses was found [108]. Sequence analysis showed that proviruses in a single cell were often genetically distinct, and gave rise to recombinants [108].

## Discussion

### Are mechanisms of SIR comparable among retroviruses?

Apart from the immune response, other cellular mechanisms are operational to prevent superinfection of cells by a second, related virus. SIR mechanisms from three retroviruses, from simple to complex, have been reviewed here. Are there any general lessons to be learned from these studies? For MuLV, a simple retrovirus that contains no accessory genes, SIR mechanisms have been deduced for two viral genes, *Gag *and *Env *that were captured by the host. Expression of these genes prevents infection of the cells by MuLV, probably by interfering with viral entry and reverse transcription. For FV, a more complex virus, the accessory gene-encoded protein Bet induces SIR, as did expression of the Env protein. The situation with HIV, the most complex retrovirus of the three, is less clear. Receptor down-modulation occurs late in infection, induced either by the Env, Vpu or Nef proteins, but this does not seem to be the principal SIR mechanism. It may instead be more important for efficient production of virions. HIV-specific, CD4-independent superinfection resistance has been described that occurs early after initial infection, but the proteins involved have not been identified conclusively. One study ruled out Vpu, Vpr and Nef, while another study showed that expression of *Nef *induced complete resistance against a challenging HIV strain, possibly by interfering with Gag processing. In the latter study, *Gag *and *Vif *expression was also found to interfere with viral replication.

Thus, no general picture regarding SIR mechanisms emerges from the study of these retroviruses. Although *Env *expression is often found to interfere with infection, simply by occupying the viral receptor, the accessory proteins play a more prominent role in complex retroviruses. Especially for HIV, the mechanism is far from clear, and multiple viral proteins may be involved. In no instances have specific host factors been identified.

### SIR and clinical HIV superinfection

The most important questions regarding HIV superinfections in a clinical sense are how often do they occur, and what are the consequences? Also, is *in vitro *research into SIR translatable into clinical practice?

Concerning the first question, if recombination is a valid viral evolutionary strategy, more HIV superinfections may occur than we detect. Two papers report transient superinfections, where after a short time of proven double infection in asymptomatic patients, only a single virus is detected later on [99,102]. If transient superinfections are common, they add to our underestimation of the phenomenon.

Ideally, superinfections should be prevented by the phenomenon of SIR as well as by the immune system. However, SIR cannot protect every target cell in an organism, as only infected cells can display SIR. Neutralizing antibodies and/or virus-specific CD8+ cell response against the first virus do, unfortunately, not seem to prevent HIV superinfection [2].

In studies of vaccinated macaques, a window period for superinfection was found. Monkeys challenged with a second SIV strain later than 10 days [109] or 4 weeks [81] after the first SIV infection, resisted superinfection, whereas all earlier challenges resulted in superinfection. In studies where the animals were challenged much later (15–122 weeks), all monkeys except one in the 15-week challenge group were resistant to superinfection, irrespective of the infection route [110-113]. However, in humans it is questionable whether a such a window period is operational, as for example in the study of Ramos *et al*. [114], one subject became infected 3–6 weeks after the initial infection, while a second patient was superinfected 5–9 months after seroconversion. Also, in the study by Yerly *et al. *[99], superinfections occurred years after the initial infection. In the chronic phase of infection, only a small fraction of susceptible cells are infected and many remain available to host a second virus. During disease progression, as CD4+ cells and the CD4 levels of infected cells decline, the patient should become less susceptible to superinfection, also because pathologic symptoms decrease the risk of re-exposure. Possibly, every patient is susceptible to HIV superinfection at some time, with the risk of re-exposure being the main limiting factor. It could also be that superinfected patients have some molecular defect that allows them to establish a second productive infection, or that the primary HIV strain is defective in SIR induction. That would imply that most HIV-infected individuals possess some resistance mechanism, and that the few identified HIV superinfected individuals among the large groups of HIV-infected participants are exceptions. However, in chronically infected patients, a productive HIV superinfection could be regarded as an opportunistic infection that warrants the diagnosis of AIDS. Here it is important to note that HIV-1 superinfection is associated with an increased viral load, a decrease in CD4+ T cell count, and increased disease progression in most cases. A shorter time to death was seen in HIV-2 dually infected monkeys compared to animals that resisted superinfection [81]. So, a productive HIV superinfection should be considered as a marker of disease progression and the start of the AIDS phase.

## Competing interests

The author(s) declare that they have no competing interests.

## Authors' contributions

ACvdK designed the review, MN and ACvdK drafted the manuscript, and BB critically revised the manuscript.
